# Comparative Study on Interaction of Form and Motion Processing Streams by Applying Two Different Classifiers in Mechanism for Recognition of Biological Movement

**DOI:** 10.1155/2014/723213

**Published:** 2014-09-03

**Authors:** Bardia Yousefi, Chu Kiong Loo

**Affiliations:** Department of Artificial Intelligence, Faculty of Computer Science and Information Technology, University of Malaya, 50603 Kuala Lumpur, Malaysia

## Abstract

Research on psychophysics, neurophysiology, and functional imaging shows particular representation of biological movements which contains two pathways. The visual perception of biological movements formed through the visual system called dorsal and ventral processing streams. Ventral processing stream is associated with the form information extraction; on the other hand, dorsal processing stream provides motion information. Active basic model (ABM) as hierarchical representation of the human object had revealed novelty in form pathway due to applying Gabor based supervised object recognition method. It creates more biological plausibility along with similarity with original model. Fuzzy inference system is used for motion pattern information in motion pathway creating more robustness in recognition process. Besides, interaction of these paths is intriguing and many studies in various fields considered it. Here, the interaction of the pathways to get more appropriated results has been investigated. Extreme learning machine (ELM) has been implied for classification unit of this model, due to having the main properties of artificial neural networks, but crosses from the difficulty of training time substantially diminished in it. Here, there will be a comparison between two different configurations, interactions using synergetic neural network and ELM, in terms of accuracy and compatibility.

## 1. Introduction

The recognition of human action is one of the interesting research field for decades in computer vision and machine learning areas. However, it has far more intriguing rout of intelligence systems and other relevant fields like psychophysical, neurophysiological, and theoretical neuroscience especially once it comes to biological movement mechanic which needs relevancy between biological and machine models. Studies in the area of physiologic and psychophysical have presented that there are several various processes for mechanism of biological motion analysis. It operates through detecting local energies in displacements of motion (see [1–3]). There are some spatial frequencies tuning considering inconsistency variations and contrast in luminance [1, 3]. In terms of motion analysis local or global motion, motion patterns have substantial influences. Temporal characteristic is considerable in perception of the movements too. Moreover, synchronisation of object features bindingly [4] along with its motion and perceiving time is also proceeded in temporal processing [5] (with respect to visual system functionality of temporal limitations [6]). Besides the aforementioned points, recognition of biological movements in mammalian visual system is considered through two separated pathways. Each of these pathways is involving certain information, that is, motion representing information of dorsal processing stream and form pathway which involves data from ventral stream.

**Figure 1:**
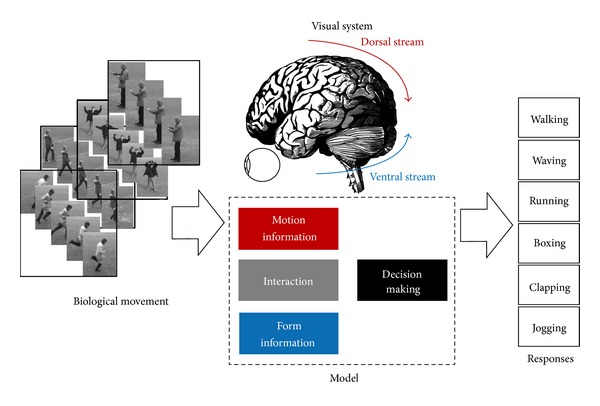
Figure reveals the analytical visual system model. The proposed approach suggests utilization of better interaction and classifier in model of the biological movement. To develop the computational models for recognition mechanism and characterize the recognition responses regarding various actions. The model is the perspective of the original model and consists of particular computations of motion and form feature data. The model operates for high-dimension of input streams and the outcome is a combination of the ventral and dorsal processing stream.

Two streams have used neural detectors for motion and form feature extraction and hierarchically allow the independency in size and style in both pathways and classification of generated features from both feed-forward pathways to categorize the biological movements. Corresponding results on the stationary biological motion recognition revealed that discrimination can be accomplished through particularly small latencies, constructing an important role of top-down unlikely signals [7]. The body shapes are determined by set of patterns like sequences of snapshots [8] which has constant feature within whole action episode. The presented method expands an earlier model used for stationary objects [8–13] recognition by adding and combining over the temporal information in pathways following the psychological evidences [14, 15]. It can be good relating to quantity tool for organizing, summarizing, and interpreting existent information based on the data provided by psychophysics, neurophysiology and functional imaging [8]. The approach quantitatively develops the original model for temporal analysis and even in computer simulations with respect to previous model architecture (see Figure 1).

Motion pathway involves information of optical flow which has fast natural temporal changes. It has consistency with neurophysiological data from neural detectors. Changing variation features due to its achievements within short changes between Frame(*t*) and Frame(*t* + 1) (*t* represents the time for each frame) creates instability in data attained from this pathway. Local detector of optical flow is connected with motion patterns and the model comprises population of four directed neurons in area of MT. However there is a connection between MT and V4 for motion and direction selection. Also, the motion edge selector cells (which have two opposite directions sensitivity) that it finds in areas of MT, MSTd, MSTl [16, 17], and many other parts of the dorsal steams and probably in the kinetic occipital area (KO) [8]. Also, motion selective edges can be like MT [16] and MSTl [17] in macaque monkey. Mild instability in the information of this pathway can be a cause of disparity in the final decision. This problem has been properly solved by applying an inference system in this pathway which substantially decreased instability throughout the fast varying pathway.

Few models have been proposed for recognition of human body shape which is plausible and neurophysiologically uses for recognizing stationary form (for instance [9]). Our method follows an object recognition model [9] which is composed of form features detectors through utilization of ABM. It follows the data obtained from neurophysiological information concerning scale, position, and sizes invariance, in case of adaptive ABM, which needs further computational load along with the hierarchy. The methods which use Gabor like filters to model the detectors have good constancy by simple cells [18]. The complex-like cells in V1 area or in V2 and V4 are invariant in terms of position varying responses (see [8]) and size independency, typically in the area of V4. V2 and V4 are more selective for difficult form features, for example, junctions and corners while being not appropriate for motion recognition because of temporal dependency in these two pathways. Snapshots detectors is used to find the shape (form) pattern similar to the area of IT (inferotemporal cortex) of monkey where the view-tuned neurons located and complex shapes tune [16]. Snapshot neurons are like view-tuned neurons in area of IT and gives independent scale and position. Previous models used Gaussian radial basis functions for modelling and it adjusted in training which performed a key frame regarding training sequences.

The final decision for recognition of biological movements is a combination of this information, the so-called interaction between two independent processing pathways. Interaction of these two streams is done at few levels in mammalian brains [19, 20] whereas many neurobiological, physiological, and psychological evidences show that the information coupling occur in many places for instance in STS level [21] and in different ways, that is, recurrent feedback loops [14]. Mutual links have suggested recurrent processing loops that permit interaction of top-down and bottom-up processing [14, 15, 22]. However, current neuroscience and psychophysics research specifies more extensive form signals influences on motion processing than previously assumed [15].

**Figure 2:**
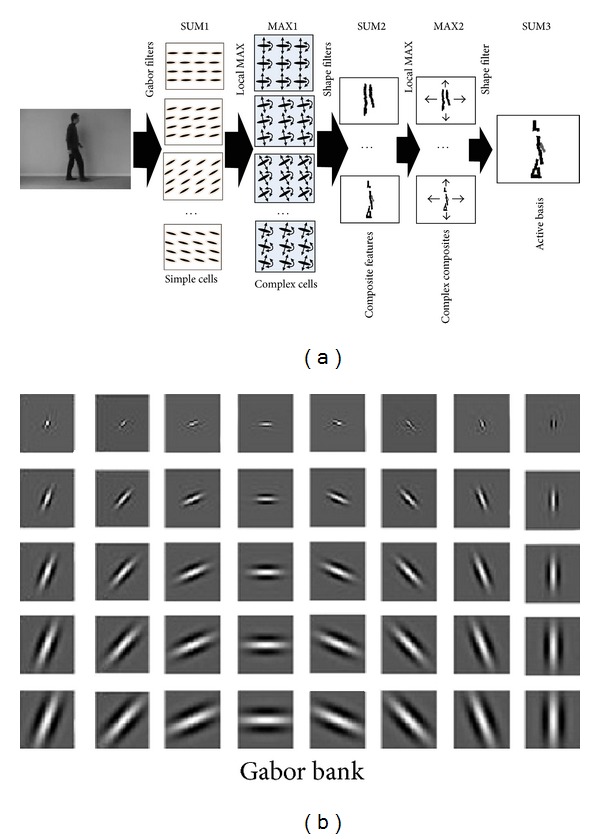
The explanation diagram of the ventral processing of the applying active basis model [24] which represents movements pattern and shape form of biological object within its movement episode. Active basis model is a Gabor based supervised object recognition method which can learn the object shape in the training stage and can be a utilized object recognizer within the action episode. (a) The processing digram of the ABM process for finding human object presented. The similarity between the method and biological finding in different level has been mentioned in different stages. (b) It represents the Gabor bank filter in different scales and orientations. Overall, ABM has two stages, SUM and MAX, which make the hierarchy from simple cells to complex cells and at the end whole human object shape by active bases.

We introduce a comparison of two different perspective models which follow the original models utilizing ABM considering the interaction portion between these processing pathways along with decision making segment. These interactions consider two different structural and inference models. Computational simulating along with testing the method is presented in the Results section. Finally, we conclude the recognition of biological movements model at the end; for examination of the proposed approach on a broader range of high-dimensional video streams, we measured responses to separated parallel pathways of visual system and overall results have been compared. Results for an instance patterns model in ventral path are revealed (Figure 2). The proposed model does a significant task of catching the constant pattern of ventral pathway responses to human movements (Figure 2, upper processing stream). The model considers the dorsal covering responses as almost half of the visual system decision The form pattern features in the model of visual system considers Gabor like stimuli in the form of hierarchical representation for object recognition task throughout the ventral stream. ABM as Gabor based supervised method can boost the responses of the stream directive and can be excellent interpreted as providing the human object. Proposed model tries to increase performance by incorporating the form features with motion features form dorsal stream and using different classifiers for this aim in original model (see [8]).

## 2. System Overview

In this paper, a comparison investigation has been addressed between two classification methods in mechanism for recognition of biological movement model. It follows the original model and considers the psychological evidences regarding the model improvement. Furthermore, decision making portion in the model has been improved that it itself increases accuracy rate and complementary part of this comparison. For this aim, additional parts to the model have been presented and afterward in-depth comparison results will be presented.

### 2.1. Active Basis Model for Form Pathway

Gabor wavelet has been previously introduced to mammalian visual system model due to similarity with its stimuli in portion; however this kind of features has been widely used for human action recognition task (e.g., [23]) and similar tasks. Gabor wavelets (in dictionary elements) provide biologically deformable templates and have been widely used by active basis model (ABM) [24]. Shared sketch algorithm (SSA) tracks through AdaBoost. Within every repetition, matching pursuit followed by SSA selects a wavelet element. The objects numbers in different orientation, location, and scale are checked by this method. Choosing the minor dictionary elements for each image (sparse coding), there can be an image representation applying linear combination of mentioned elements by considering *U* a minor residual. SSA interacts with information of motion pathway and visually guides:

(1) $$\begin{array}{c} I=\overset{n}{\underset{i=1}{\sum }}c_{i}\beta_{i}+\epsilon . \end{array}$$

Let *β* = (*β*_*i*_, *i* = 1,…, *n*) be Gabor wavelet set of sinusoid elements and components, *c*_*i*_ = 〈*I*, *β*_*i*_〉, and *ϵ* an image coefficient which is kept unsolved [24]. Using wavelet sparse coding a large number of pixels reduce to small number of wavelet element. Training the natural image patches through sparse coding can be executed by dictionary elements of Gabor like wavelet which carries the simple cells in V1 [25]. Local shape extraction will be discretely done for entire frames similar to [24] filter responses in density and orientation for each pixels. ABM uses Gabor filter bank but in different form. A dictionary of Gabor wavelets contains *n* directions and *m* scales in the form of GW_*j*_(*θ*, *ω*), *j* = 1,…, *m* × *n*, where *θ* ∈ {*kπ*/*n*, *k* = 0,…, *n* − 1} and $\omega =\{\sqrt{2}/i,i=1,\ldots ,m\}$. Features of Gabor wavelet specify the posture, size, and location small variance of object form. In overall shape structure is considered to be maintained during the recognition process. Every element response (convolution) offers the information of form with *θ* and *ω*. Consider

(2) B=〈GW,I〉=∑∑GW(x0−x,y0−y:ω0,θ0)I(x,y),

where GW_*j*_ is a [*x*_*g*_, *y*_*g*_], *I* is a [*x*_*i*_, *y*_*i*_] matrices, and response of *I* to GW is a [*x*_*i*_ + *x*_*g*_, *y*_*i*_ + *y*_*g*_]. Consequently, earlier both matrices convolution must be expanded by adequate zeroes. Convolution consequence can be removed via the result gathering. An extra technique would be to shift back the frequencies centre (zero frequency) to the image center although it might be considered loosing data reason. Training set of image shown by {*I* ^*m*^, *m* = 1,…, *M*}, SSA consecutively chooses *B*_*i*_. The important opinion is to find *B*_*i*_ and thus the segments edges attained from *I*_*m*_ become maximum [24]. It requires to calculate [*I* ^*m*^ · *β*] = *ψ* | 〈*I* ^*m*^ · *β*〉|^2^ for different *i* where *β* ∈ Dictionary and *ψ* signifies sigmoid, whitening, and thresholding transformations. Then for maximizing [*I* ^*m*^ · *β*] for all possible *β* will be computed, where *β* = (*β*_*i*_, *i* = 1,…, *n*) is the template, for every training image *I* ^*m*^ scoring will be based on

(3) $$\begin{array}{c} M\left(I^{m},\theta \right)=\overset{n}{\underset{i=1}{\sum }}\delta_{i}\left|I^{m},\beta \right|-\log\Phi \left(\lambda \delta_{i}\right). \end{array}$$

*M* is function of match scoring and *δ*_*i*_ attained from ∑_*n*=1_^*M*^[*I* ^*m*^, *β*] concerning steps selection, and Φ is nonlinear function. The exponential model for logarithmic likelihood relation is attained from the template matching scores. The weight vectors are calculated by technique of maximum likelihood and are exposed by Δ = (*δ*_*i*_, *i* = 1,…, *n*) [24]. Consider

(4) $$\begin{array}{c} \mathrm{Max}\left(x,y\right)=\underset{(x,y)\in D}{\max}M\left(I_{m},\beta \right). \end{array}$$

*Max*⁡(*x*, *y*) computes the maximum matching score previously obtained and *D* signifies *I* lattice. Here, there is no summation because of updating the size based on training system on frame (*t* − 1). Moreover, method tracks the object relating to motion feature to signify the moving object displacement. These displacements have been assisted to be detected better through guidance of motion information which is considered a substantial similarity with biological evidences [14, 15, 25].

### 2.2. Dorsal Pathway and Motion Information

The information of motion in recognition of biological movements is attained using optical flow. It finds out the movement pattern which has reliability by information of neurophysiological from neural detectors hierarchy. Areas of V1 and MT have some neurons for motion and direction selection in initial motion pathway level correspondingly. Visibility of every layer shows the principle dissimilar between previous and layerwise optical flow estimation. Shape of mask can perform while matching applies for the pixels which fall inside the mask (see [26]). Applied layerwise optical flow method (mentioned in [26]) has baseline optical flow algorithm of [27–29]. In overview, *M*_1_ and *M*_2_ are visible masks for the two frames *I*_1_(*t*) and *I*_2_(*t* − 1) and the fields of flow from *I*_1_ to *I*_2_ and from *I*_2_ to *I*_1_ are denoted by (*u*_1_, *v*_1_) and (*u*_2_, *v*_2_). Following terms will be deliberated utilizing the layerwise optical flow estimation. Objective function contains summing three parts and visible layer masks match these two images using Gaussian filter which called data terms matching *E*_*γ*_^(*i*)^, symmetric *E*_*δ*_^(*i*)^, and smoothness *E*_*μ*_^(*i*)^. Consider

(5) $$\begin{array}{c} E\left(u_{1},v_{1},u_{2},v_{2}\right)=\overset{2}{\underset{i=1}{\sum }}E_{\gamma }^{\left(i\right)}+\rho E_{\delta }^{\left(i\right)}+\xi E_{\mu }^{\left(i\right)}. \end{array}$$

After objective function optimization and applying inner and outer fixed-point repetitions, coarse to fine search, bidirectional flows are attained and utilized for specifying the motion patterns. Compressed optic flow for all frames is calculated by straight template matching earlier frame applying the absolute difference summation (L1-norm). Though optic flow is principally noisy, no smoothing techniques have been done on it as the field of flow will be blurred in gaps and specially the places where motion information is significant. To get the proper optical flow response about its application in recommended model, optical flow will be used for adjusting active basis model and making it more efficient. To attain a reliable illustration through form pathway, optic flow estimates the velocity and flow direction. Response of the filter based on local matching velocity and direction will be maximal as these two parameters are constantly changing.

### 2.3. Fuzzy Inference in Dorsal Processing Stream

Fuzzy logic is a multivalued logic, that is, created from fuzzy set theory found by Zadeh (1965), and it deals with reasoning approximation [30]. It delivers great framework targeted at approximation reasoning which can suitably bring the imprecision and uncertainty together in model expert heuristics and linguistic semantics and handles necessary level organizing principles. A time dependent fuzzy system also uses many times regarding solution of control and classification and so forth, Chen and Liu (2005) present a delay-dependent robust fuzzy control for a class of nonlinear delay systems via state feedback [31].

Applying fuzzy inference system involves the interaction between both pathways. A fuzzy inference system to imply optical flow within motion pathway has been presented by considering the flow in every frame division and estimation of the membership value for every portion. The problem statement through initial assumptions for the human object velocity associates for both *x* and *y* directions. In general, *v*_*x*_, *v*_*y*_ ∈ *R* ^*m*×*n*^ where *m* and *n* are sizes of image frame from input video stream.

Membership functions in triangular shapes for *v*_*x*_ and it will be the same for *v*_*y*_ velocity in *x* and *y* directions and signify quaternion correlator outputs in the enrolment stage belonging to motion pathways, respectively (i.e., *μ*_*v*_*x*__^*C*_1,2_^(*x*), *μ*_*v*_*x*__^*C*_2,4_^(*x*), *μ*_*v*_*x*__^*C*_1,2_^(*y*), and *μ*_*v*_*x*__^*C*_2,4_^(*y*)) [32]. Maximum velocity in two coordinates has been considered for estimation of membership values which are related to each cell. Aggregation of these values will be considered and helps in overall judgement throughout the sequential frames within the path. The dependency regarding time variation for every frame of video in this pathway is through definition of fuzzy membership scoring for every time division. Velocities information can be unstable due to many environmental situations, for example, camera shaking, dissimilar style of human object temporarily in front of camera, and the velocity amount being time dependent. Time definition in this context is based on the frame time per second and creates resistance for every frame with respect to previous score value of membership. It can be involved in mathematical parameter or even just additional programing algorithm.

Time dependent fuzzy optical flow division can be used for signifying an optical flow divisions class with fuzzy inference rules in time for every frame of video stream as unit of time defined here, as follows:

(6) μ~vCi(t)=μ~vCi(t−τ)+ηvCi(t)(1−μ~vCi(t−τ))t∈[t0,t0+kτ],  k∈(0,1,…,N),

where *τ* is the frame time which is a parameter for camera and *k* is numbers of frames pasted from the cell changing (it means *k* will be reset after varying the cell membership). *N* is the maximum number of frame distance from present frame which does not unreasonably increase membership function value. We call *η*_*v*_^*C*_*i*_^(*t*) memory coefficient function and it can be just a mathematical parameter or programming algorithm to add the winner cell membership. *t* is the frame time where one division optical flow has the highest membership amount as compared with other divisions and it will be restarted by changing the division. At the end, aggregation of fuzzy inference scoring for flow in different body has been computed. Defuzzification has been done through IF-THEN rule and output belongs to highest scores among the actions classes and specifies the movement. The max. amount represents degree of belonging to each classes and at the end the decision will be based on “winner takes all” (selection of the maximum). For example, running, jogging, and walking involve the lower limb activities whereas boxing, clapping, and waving make flow in the upper limb of human object (for interested readers, please refer to [32]).

### 2.4. Extreme Learning Machine (ELM)

Neural networks have been widely utilized in several research areas because of their ability to estimate difficult nonlinear mappings straight from the sample of input as well as offering models for a big class of artificial and natural phenomena that are problematic to hold via classical parametric techniques. Recently, Huang et al. [33–35] presented a novel algorithm for learning regarding single layer feed-forward neural network structural design named extreme learning machine (ELM) that solves the problems initiated through algorithms using gradient descent, for instance, backpropagation used in ANNs. ELM is able to considerably diminish the time quantity required to train neural network and has greatly enhanced faster learning and generalization performance. It needs lesser interventions of human and can run significantly faster than conventional techniques. It routinely concludes the parameters of network entirely, which evades unimportant external intervention by human and more effective in real-time applications. Some advantages of extreme learning machine can be named: simplicity in usage, quicker speed of learning, greater generalization performance, appropriateness for several nonlinear kernel functions, and activation function [36]. Single hidden layer feed-forward neural network (SLFN) function with hidden nodes [37, 38] can be shown by mathematical explanation of SLFN integrating additive and sigmoid hidden nodes together in a joined method provided as follows:

(7) $$\begin{array}{c} f_{L}\left(x\right)=\overset{L}{\underset{i=1}{\sum }}\beta_{i}G\left(s_{1},b_{i},x\right),\;x\in R^{n},\;\;a_{i}\in R^{n}. \end{array}$$

Let *a*_*i*_ and *b*_*i*_ be the parameters of learning in hidden nodes and *β*_*i*_ represent the connecting weight of *i* ^*t*^ *h* for output node of hidden node. *G*(*s*_1_, *b*_*i*_, *x*) is the output of *i* ^*t*^ *h* hidden node with respect to the input *x*. For additive hidden node with activation function *G*(*x*) : *R* → *R* (e.g., sigmoid and threshold), *G*(*s*_1_, *b*_*i*_, *x*) is given by

(8) $$\begin{array}{c} G\left(a_{i},b_{i},x\right)=g\left(a_{i},x+b_{i}\right),\;b_{i}\in R. \end{array}$$

Let *a*_*i*_ be the connecting weight vector of the input layer to *i* ^*t*^ *h* hidden node and *b*_*i*_ the *i* ^*t*^ *h* hidden node bias. For *N*, arbitrary different examples (*x*_*i*_, *t*_*i*_) ∈ *R* ^*n*^ × *R* ^*m*^. Now, *x*_*i*_ is a *n* × 1 vector of contribution and *t*_*i*_ is a *m* × 1 vector of target. If an SLFN by *L* hidden nodes can be estimated, these *N* samples have zero error. If then infers that there exist *β*_*i*_, *a*_*i*_, and *b*_*i*_ such that

(9) $$\begin{array}{c} f_{L}\left(x_{j}\right)=\overset{i=1}{\underset{L}{\sum }}\beta_{i}G\left(a_{i},b_{i},x\right),\;j=1,2,\ldots ,N. \end{array}$$

The equation above is mentioned in compacted way as follows:

(10) $$\begin{array}{c} H\beta =T, \end{array}$$

where

(11) $$\begin{array}{c} H\left(\hat{a},\hat{b},\hat{x}\right)={\left[\begin{array}{cc} G\left(a_{1},b_{1},x_{1}\right) & G\left(a_{L},b_{L},x_{1}\right)\\ G\left(a_{1},b_{1},x_{N}\right) & G\left(a_{L},b_{L},x_{N}\right) \end{array}\right]}_{N\times L}, \end{array}$$

with $\hat{a}=a_{1},\ldots ,a_{L}$; $\hat{b}=b_{1},\ldots ,b_{L}$; $\hat{x}=x_{1},\ldots ,x_{N}$. Consider

(12) $$\begin{array}{c} \beta ={\left[\begin{array}{c} \beta_{1}^{T}\\ \vdots \\ \beta_{L}^{T} \end{array}\right]}_{L\times m},\;\;T={\left[\begin{array}{c} t_{1}^{T}\\ \vdots \\ t_{L}^{T} \end{array}\right]}_{N\times m}. \end{array}$$

Let *H* represent the hidden layer of SLFN output matrix with *i* ^*t*^ *h* column of *H* being *i* ^*t*^ *h* hidden nodes output with respect to inputs *x*_1_, *x*_2_,…, *x*_*N*_. In terms of method application, the proposed approach seems to be a straight video processing task for machine. The rate of involving video frame is very much dependent on temporal order considering the information extraction in each pathway. ABM requires two frames by having two time unit differences and it is very similar with motion pathway. Considering that there will be an implementation of interaction between two independent processing streams which comprises the visual guidance from optical flow to SSA which it needs more frames, it means every frame for being processed by ABM involves two frames for motion information. Furthermore, ABM itself requires two frames for operation so generally four frames are needed to operate whole system for one step. However, in the case of no internal additional interaction, there will be just two frames for each step.

## 3. Experimental Results

The approach has considered recognition task of biological movement in mammalian visual system. It followed the original model in this area whereas it has been scrutinizingly developed in many parts including process of object recognition in the form pathway and implying fuzzy inference in motion pathway. Yet, development in this model has suggested the implementation of interaction within both pathways processing streams. However, in the comparison part, both cases have been investigated. In addition, the influence of various classifiers for changing the decision making portion also has been analyzed. Two different classifiers have classified to examine the decision making effects in the model. Besides all these biologically inspired explanations, this machine perspective of the task is human action recognition. There must be many important cautions to be considered, including the biological point of view during entire steps of the task. For benchmarking of the method and following computer vision normality and estimation of the accuracy and performance, human action recognition datasets have been used. For general accuracy of system performance, KTH human action [39] has used and comparisons have been recorded and presented in the following sections. Moreover, Weizmann human action recognition robustness dataset [40] is also used to show the robust performance using the presented techniques. KTH action dataset as one of the principal single person human action datasets contains 598 action sequences and six different single person actions types, that is, boxing, jogging, clapping, walking, running, and waving. 25 people perform the actions in diverse conditions: outdoors with different clothes (s3), outdoors with scale variation (s2), outdoors (s1), and indoors with lighting variation (s4). Here, the sequences resolutions become 200 × 142 pixels through downsampling. For the approach, 5 random cases (subjects) have been used for training and making the form and motion predefined templates. As it is mentioned in the literature, KTH is a robust intrasubject difference with large set whereas the camera for taking the video throughout the preparation had some shacking and it creates many difficulties to use this database. Furthermore, it has four scenarios which are separately and independently tested and trained (i.e., four visually different databases, which share the same classes). Both alternatives have been run. For considering the human actions symmetry problem, there is a sequences mirror function along with vertical axis which can be obtainable for testing and training sets. Here all probable human actions intersection has been considered (e.g., one video has 24 and 32 action frames.)

### 3.1. Contribution between Motion and Form Features

Substantial contribution on the model development insipid of supervised Gabor wavelet based object recognition and additional inference fuzzy system in motion processing pathway is considered as interaction of two processing pathways along with utilization of different decision making part that can be done through changing the classifiers and analysis of its performance. Considering that there are many ways for combination of information obtained by these two processing pathways and much psychological, physiological, and neurophysiological evidence regarding the interaction of independent processing streams, this approach follows the mentioned valuable evidence to improve previously presented models (all follows the recognition mechanism of biological movement in original model). Importance of this interaction between these pathways is investigated via benchmarking performance within state-of-the-art methods (please see Table 1). Furthermore, the comparison is not only valuable in terms of information interaction decision making performance. It has very substantial result to represent the performance of modification in decision making parts. Here, we have shown a method for development of biologically inspired model of biological movement with respect to the original model and previous approaches. Feature extraction for pathways interaction and decision making between them has been considered which modified the feed-forward structure of these independent information.

### 3.2. Results

Implementing our method in terms of accuracy is considered as two stages for the general accuracy and stability test. The general accuracy is obtained for comparison study for interaction justification within the processing streams that have been done using KTH human action recognition dataset. For this aim comparison with state-of-the-art methods also considered the same dataset. Task of the proposed method has been implied by general human action recognition task. However, this task was also the same in the stability testing. Weizmann human action robustness dataset is used concerning the cluttered background benchmarking of robustness. Using ABM is one of the strength points of proposed development in the model. Furthermore, optical flow involvement and information combination between two processing pathways can be a very good reason for this. Fuzzy inference system in the motion pathway is a good point for increasing the robustness. It is very obvious due to eliminating very quick changes of flow within optical flow outcomes. Fast variations of flow in motion pathway usually can be a cause of disparity within the decision making processes. This can diminish accuracy rate for the model and it is not realistic in the actual environmental situations because every second of the video including many frame images and changing the action in fraction of second and within the frames seems far from reality. It must be considered because the model is the implementation of mammalian visual system. An overview to attain the action prototype way and its discussion is considered in this portion. The comparison of development in the approach in the aspect of interaction along with decision making expansion is illustrated and discussed in this section.

### 3.3. Overview on Action Prototypes

As it is used and presented [32, 41], every human action has certain form similarity and specific structural configurations. These mentioned shapes can be a substantial abstract of every human action during time process in video. We divide every human action movement in its sequences to five primitive basic movements which is not necessarily common among various movements. These primarily action abstracts are called action prototypes and can mostly reconstruct every human action applying them. They also can be very good representative of the action in many environmental situations and style invariance property in the actions. It is motivated by the training map of human objects within the actions or any other tasks. These action prototypes have been computed through two-time utilization of synergetic neural network melting for every different action which gives action abstracts. For this aim five different action episodes are randomly chosen and considered as training map of the proposed approach and excluded from the testing dataset. Deliberate prototype images seeing style invariance signify one action in five different snapshots (for more information please refer to [32, 41]). The outcomes of melting process in synergetic neural network does very much look like abstracting a set of human object actions using eigendecomposition which gives eigenimages within a set. The action prototype has a very significant and essential role in the form processing information in the ventral stream.

### 3.4. Experimental Results

The benchmarks are mentioned in this section and the approach follows the implementation of fuzzy inference using optical flow division presented in [13] and further interactions are scrutinizingly investigated. Moreover, modification in decision making section explores in-depth. The task in this section considered more look-alike computer vision task regarding human action recognition. Confusion matrices are obtained in the similar experimental conditions as [13]. The tables and confusion substantially represent the better result presentation within modification of the classifier and decision making block of the algorithm in biologically inspired model. Recognition accuracy comparison has been demonstrated in Figures 6 and 7. Comparison performed by depiction of the accuracy in terms of comparison with state-of-the-art methods and similar methods which are more biologically inspired. Similarity of the presented model has been deliberated in the assessment. KTH human action dataset is used for benchmarking and the evaluation assessment compared with state-of-the-art methods in the same dataset for consistency in the experimental results (see Figure 4) [11, 42–46]. Also it is noticeable, as previously mentioned, that the training map and action prototypes obtained from the random selection of the human action set in four different scenario videos from KTH and excluded from the testing set have no overlap between these two sets. Utilization of the training map within the performance estimation is shown by simple comparison in current videos frames snippets with the action prototype which is merely template matching. It gives a score of the matching for every human action prototype. It comprises the information of form representing the ventral processing streams outcomes and needs to involve the information motion pathways.  Figure 6 depicts the confusion matrix and Figure 7 shows some results of the proposed expansion in the recognition mechanism of biological movements. Confusion matrix rows denote the results of corresponding classification, although, respectively, columns signify the examples to be classified. As it is shown through these figures and corresponding results, the highest confusion happens among running, jogging, and walking. To distinguish these actions is difficult as the actions performance by some subjects has resemblance. Correspondingly, another misclassification happens mainly between alike classes, similar earlier confusion, or hand-clapping and hand-waving (please see confusion matrices in Figures 5 and 6). Following the mentioned parts regarding the action prototypes computed by twofold synergetic neural network melting within whole action frames. These action prototypes perform as action abstract within the recognition mechanism. It can be used for recognition and categorization of action in the form pathway. Besides, there was an adjustment in this pathway which involved the motion information into form path via analysis of the type of action whether it occurs in lower or upper limb; the relevant membership function organized this task. There can be a discussion for this performance; this approach implementation can be done through a simple programming rather than complex mathematical computation. The method gives very good time delay memory which is totally time dependent and it provides robustness within quick changes of optical flow and motion information plus dramatically diminishing the disparity rate.

### 3.5. Relation to Existing Methods and Discussion

**Figure 3:**
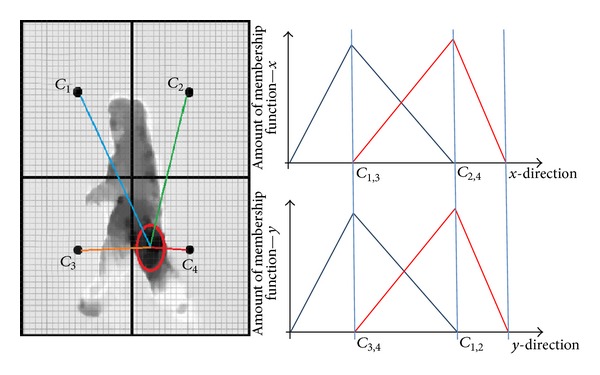
The result of dorsal processing stream applying optical flow [26] and the optical flow division into the fuzzification has been depicted. The resolution of divisions is designed for categorization of actions group to have additional interference of dorsal and ventral processing streams. It can be a good representative of the interaction on MT, middle temporal of dorsal stream, and V4, ventral stream (for shape and orientation), or the MST area with inferior temporal (IT) (see more details in [14, 15]). The membership function of the action will be estimated from the position of maximum flow in the flow image. Membership values are aggregated through the proposed technique to increase the robustness. The input image of action mentioned in the figures is obtained from KTH human action recognition dataset [39].

**Figure 4:**
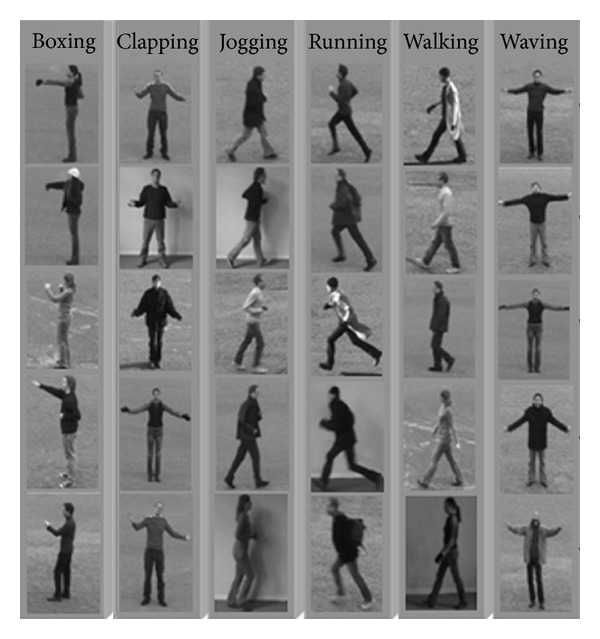
The figure depicts KTH human action dataset. To test the recognition of biological movements one of the well-known human action recognition datasets has been utilized in its performance. Here, the set represents KTH human action dataset. It is noticeable to mention that KTH dataset is one of the largest human action datasets having six various human actions in four different scenarios.

**Figure 5:**
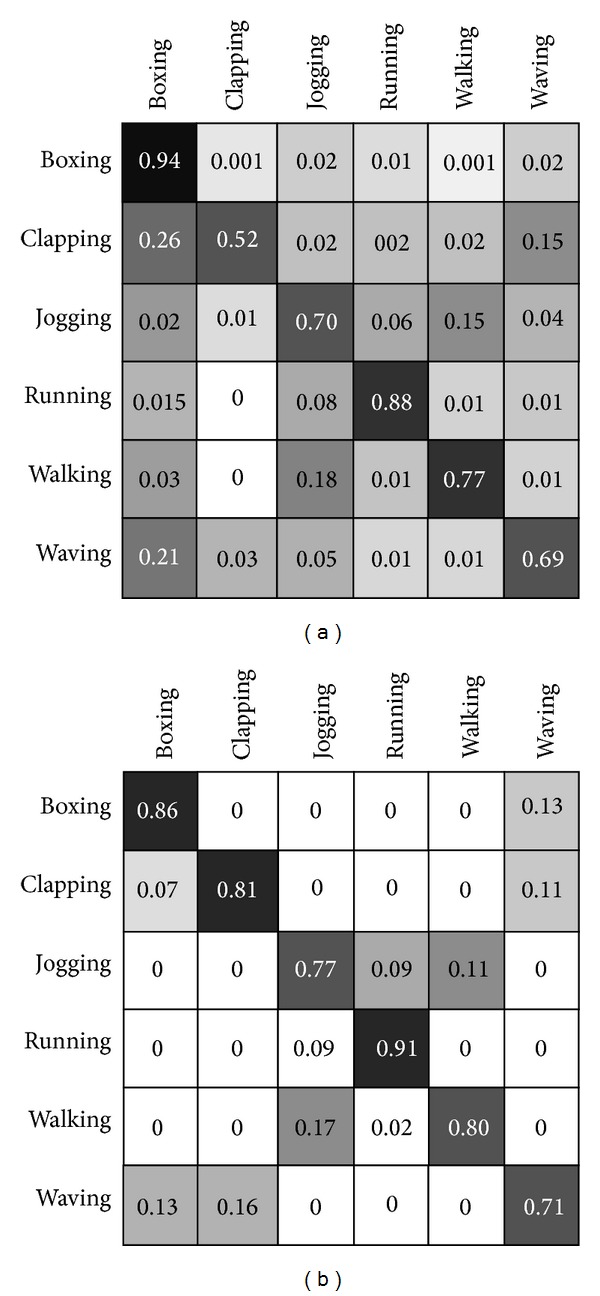
Confusion matrices SNN classifying KTH dataset obtained by adapted active basis model as combination of form and motion pathways. Confusion matrices of the proposed approach have been presented for the case without fuzzy interference system, left matrix, and, after it, right matrix which are achieved from human action movements of KTH dataset [39]. The robustness of the method after adding the fuzzy interference stabilizer is considerably increased. The wrong recognitions in the left confusion matrix have been decreased especially in case of some actions, that is, clapping. Moreover, soar of robustness helps increase the overall accuracy and gives better results in classification of biological movement. The accuracy of categorizations using unbalanced SNN is reached at 86.46%.

**Figure 6:**
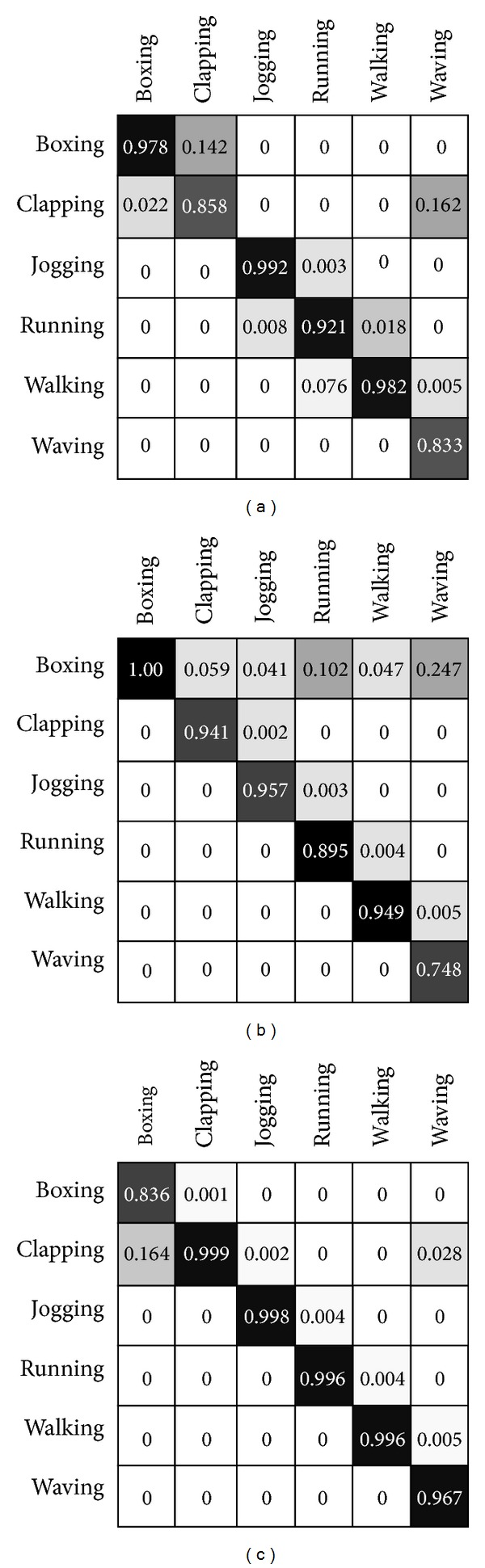
Confusion matrices ELM classifying KTH dataset attained by adapted active basis model as combination of form and motion pathways. Confusion matrices of the proposed approach have been presented which is obtained from human action movements of KTH dataset [39]. There are three different kernels which have been used in classifying using ELM algorithm [33–38] in the decision making and categorization of the biological movement. From left to right, RBF kernel-ELM, wavelet kernel ELM, and sigmoid-ELM confusion matrices have been depicted where sigmoid kernel-ELM has better results in classification of biological movement. The accuracy of categorizations is ELM-Wav = 91.5%, ELM-RBF = 92.7%, and ELM-Sig = 96.5%.

**Figure 7:**
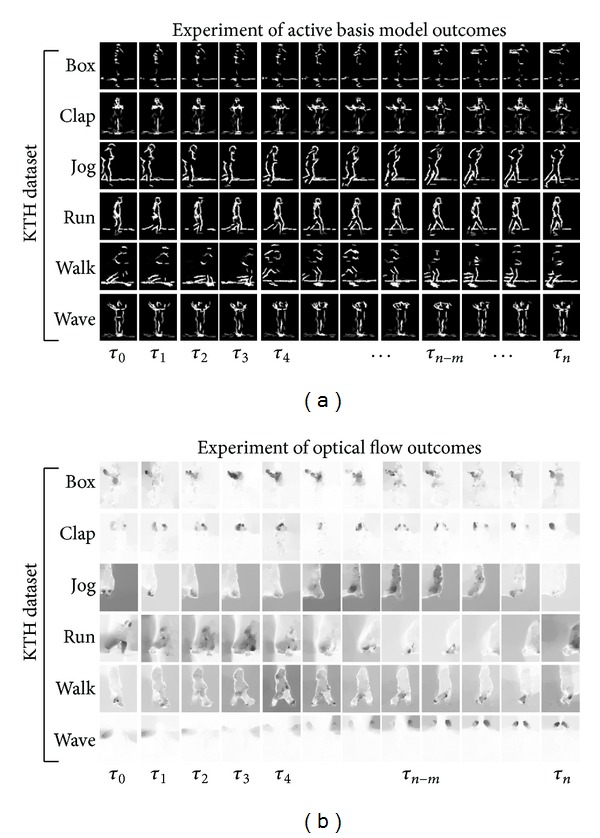
Simulation results for simple biological movement paradigm based on ABM [24] in the ventral processing stream and optical flow [26] in dorsal stream are shown. Each row within the panel reveals the response of ABM during the episode as well as flow generated for every different action. The set of biological movements belongs to the biological movements which is from KTH dataset [39]. (a) The simulation results of the different actions of KTH dataset. (b) Optical flow simulation results.

The presented method is utilized for mechanism of biological movement and main focus of this approach concentrates on interaction of two visual processing streams and decision making within these paths. Here, general difference and similarity between existing methods and this approach are shortly investigated. The method is totally in direction of psychological and physiological evidences. It particularly follows the original model of biological movement recognition [8, 11, 42, 47] considering psychological evidences [14, 15]. The obvious change in this area can be considered applying a supervised Gabor wavelet based object recognition method in ventral stream which is presented [41]. Applying ABM increases the focus of form pathway in information of form and structure of human object and provides more stability in the recognition task. Moreover, it follows the characteristic of simple and complex cells to attain the shape of object in form pathway and gives reliability and robustness in form pathway particularly in the clutter background. On the other hand, information of motion is considered through utilization of optical flow in dorsal pathway. Optical flow can substantially give motion information within the video frames and object movements can be shown by simple silhouette representing the flow of human object within the considered frames. Optical flow is successfully used by the original model many times but it can reveal instability due to fast variation of the input video streams. The fuzzy optical flow division has been introduced for this pathway and increases the rate of stability and more reliability via delivering the time memory and time delay, in the processing of quick variation input [32]. It gives good combination of fast variation of motion information and this delay gave more robustness in the recognition mechanism. The interaction of these two parallel independent processing streams has been investigated for many years in different areas especially psychology and physiology. In visual system, Gabor like filters mainly have a representation role for simple and complex cells. ABM is an appropriate characteristic for this part, particularly concerning its contribution in object recognition task. It could follow the concerning encoded object shape [15]. The shape of object concerns in form pathway and ventral processing stream has been properly deliberated based on training phase and explanation for its reliability is done human prototypes. ABM is someway contemplate Gabor action inducement for pin-down form processing at two-level local information around limb angle from orientations and global body structure of Gabor signaled by Gabor paths spatial arrangement. On the contrary, optical flow used for motion information extraction has tracked the second characteristic and contains filtering through direction selection sensors and its incorporation for resolving the well-known aperture problem. Motion information shows both motion signals local velocity categories and motion trajectories joint utilizes signals in form path by guiding SSA in ABM [48] as a good representation of crossconnection between V4 and MT [14, 15], that is, a very substantial interaction effect within these two processing pathways [32, 41]. However, the interaction in both processing pathways is not limited to this interaction and will occur in different regions of visual processing stream. Form and motion processing principal view in human visual system, it is assumed that these two traits are controlled by self-determination and distinct modules ([8, 11, 13, 23]). It has been identified that form signal information can influence motion processing more broadly than earlier believed (see [15]) and the proposed approach reflects direct motion information effect on form processing. Visual system connectivity is categorized by crossconnections with respect to feed-forward of parallel connection ([14, 49, 50]). Optical flow division method delivers bottom-up and top-down processing interaction and connection among brain regions within dual computational streams and can be decent descriptive connection between dorsal and ventral streams (i.e., V4 and MT; see Figure 3) [14, 15]. Dorsal stream is correspondingly supposed to preform spatial computation correspondences (where) and ventral stream regarding object recognition task (what) in the cortical areas of V4, V2, V1, and IT(inferotemporal cortex) accompanied by existing conflict evidence to a whole separation of “what” and “where” in macaque brain information (see [50, 51]) demonstrating about information for position and size of objects are similarly signified in macaques inferotemporal cortex. However, proposed method is an initial spatial configuration and distinctiveness isolation into distributed processing pathways requires weighty hardware computation. However having optical flow low resolution divisions (four alienated portions) could be a worthy factor aimed at the computational load diminishing. The precise classified sequences are described as highest results existing in the field literature. To place suggested technique in this context, we have mentioned it with the state-of-the-art methods. Our method is a frame-based which tracks for all frames inaction sequences. The individual labels formerly attained from training map basically associate with a label sequence done majority voting (like [11, 13]). The interacted approach comparison by state-of-the-art methods has been performed and it is shown in Table 1. Its accuracy just represents the concerns in comparison with other similar methods indicating relative compatibility and significant performance for proposed approach.

## 4. Conclusion

The presented approach has addressed a very substantial interrelevant comparison of the interaction of two processing streams of mammalian brain visual system. The developments in decision making portion along with a significant comparison within these pathways have been scrutinizingly investigated. Generally, the interaction of motion information to form processing pathways has shown a very good and reasonable effect in the recognition model and it can represent crossconnection of V4 and MT in brain [23]. The human action prototype outcomes using twofold synergetic neural network melting have been reviewed and considered for recognition of form information in form processing pathway. For benchmarking, the task has been converted to a computer vision and human action recognition and two datasets have been used regarding evaluation and recognition performance with the state-of-the-art methods. The crossconnection in feed-forward biologically inspired method also has been presented accordingly. Correspondingly it had respectable performance in dissimilar datasets along with reasonable computational cost. As a limitation, it currently has no mechanisms for invariance alongside rotation and variations in viewpoint although it can be considered to put mechanism regarding multiscale. ABM is a delicate algorithm and requires further attention though its training still can be more developed to be a powerful tool for form pathway that is far from this approach purposes.

## Figures and Tables

**Table 1:** The proposed comparison method recognition results revealed among previous human action recognition method accuracies (bio- or non-bioinspired) on KTH human action dataset.

Methods	Accuracy (%)	Years
Schüldt et al., [39]	71.72	2004

Niebles et al., [45]	83.33	2008

Jhuang et al., [42]	91.79	2007

Schindler and Van Gool [11]	92.79	2008

Wang and Huang [34]	91.29	2005

Zhang and Tao [43]	U-SFA: 86.67	2012
	S-SFA: 86.40	
	D-SFA: 89.33	
	SD-SFA: 93.87	

Yousefi and Loo [32]	SNN: 86.46	2014

Proposed method	ELM: 96.5	
